# Soy isoflavones and their metabolites modulate cytokine-induced natural killer cell function

**DOI:** 10.1038/s41598-019-41687-z

**Published:** 2019-03-25

**Authors:** Thomas A. Mace, Michael B. Ware, Samantha A. King, Shannon Loftus, Matthew R. Farren, Elizabeth McMichael, Steven Scoville, Connor Geraghty, Gregory Young, William E. Carson, Steven K. Clinton, Gregory B. Lesinski

**Affiliations:** 1 Division of Gastroenterology Hepatology Nutrition, The Arthur G. James Cancer Hospital and Richard J. Solove Research Institute, Comprehensive Cancer Center, The Ohio State University, Columbus, OH USA; 2 Department of Internal Medicine, The Arthur G. James Cancer Hospital and Richard J. Solove Research Institute, Comprehensive Cancer Center, The Ohio State University, Columbus, OH USA; 3 Division of Medical Oncology, The Arthur G. James Cancer Hospital and Richard J. Solove Research Institute, Comprehensive Cancer Center, The Ohio State University, Columbus, OH USA; 4 Division of Surgical Oncology, The Arthur G. James Cancer Hospital and Richard J. Solove Research Institute, Comprehensive Cancer Center, The Ohio State University, Columbus, OH USA; 5 Department of Surgery, The Arthur G. James Cancer Hospital and Richard J. Solove Research Institute, Comprehensive Cancer Center, The Ohio State University, Columbus, OH USA; 6 Biomedical Sciences Graduate Program, Medical Scientist Training Program, The Arthur G. James Cancer Hospital and Richard J. Solove Research Institute, Comprehensive Cancer Center, The Ohio State University, Columbus, OH USA; 7 Center for Biostatistics, The Arthur G. James Cancer Hospital and Richard J. Solove Research Institute, Comprehensive Cancer Center, The Ohio State University, Columbus, OH USA; 8 Department of Hematology and Medical Oncology, Winship Cancer Institute of Emory University, Atlanta, USA

## Abstract

Soybeans are a rich source of isoflavones that have been linked with anti-inflammatory processes and various health benefits. However, specific mechanisms whereby soy bioactives impact immune cell subsets are unclear. Isoflavones, such as genistein and daidzein, are metabolized by microbes to bioactive metabolites as O-desmethylangolensin (O-DMA) and equol, whose presence has been linked to health benefits. We examined how soy isoflavones and metabolites impact natural killer (NK) cell signaling and function. We observe no impact of isoflavones on viability of healthy donor peripheral blood mononuclear cells (PBMCs) or NK cells, even at high (25 µM) concentrations. However, pre-treatment of PBMCs with physiologically-relevant concentrations of genistein (p = 0.0023) and equol (p = 0.006) decreases interleukin (IL)-12/IL-18-induced interferon-gamma (IFN-γ) production versus controls. Detailed cellular analyses indicate genistein and equol decrease IL-12/IL-18-induced IFN-γ production by human NK cell subsets, but do not consistently alter cytotoxicity. At the level of signal transduction, genistein decreases IL-12/IL-18-induced total phosphorylated tyrosine, and phosphorylation MAPK pathway components. Further, genistein limits IL-12/IL-18-mediated upregulation of IL-18Rα expression on NK cells (p = 0.0109). Finally, *in vivo* studies revealed that C57BL/6 mice fed a soy-enriched diet produce less plasma IFN-γ following administration of IL-12/IL-18 versus control-fed animals (p < 0.0001). This study provides insight into how dietary soy modulates NK cell functions.

## Introduction

Soy is a rich source of multiple classes of bioactive components with isoflavones (primarily genistein and daidzein) receiving considerable attention in regards to the inhibition of inflammation and cancer prevention^1^. It is hypothesized that bioactive phytochemicals in fruits and vegetables contribute to their health benefits, yet specific mechanisms often remain enigmatic. Studies to elucidate how these bioactive components impact immune modulation are challenging due to the complexity of collaborating immune cells and their intricate communication and regulatory processes. Furthermore, investigators increasingly appreciate the enormous inter-individual variability in soy isoflavone metabolism due to host processes as well as the gut microbiota^2^. For example, in some individuals, daidzein can be processed into its secondary metabolites, O-desmethylangolensin (O-DMA) and equol. It is proposed that this process is impacted by the presence of particular gut bacteria and their functional capabilities that vary for uncertain reasons among individuals. In various human populations it is estimated that approximately 30–50% of individuals have the ability to produce equol upon ingestion of soy, while 80–95% favor production of O-DMA^3^. We have previously observed that men with prostate cancer consuming a soy isoflavone-enriched bread experienced a change in circulating immune regulatory cytokine profiles consistent with a reduction in pro-inflammatory processes and immunosuppressive cell populations^2,4^. These data provide evidence for the immunomodulatory impact of soy isoflavones in a clinically-relevant setting.

Specific soy isoflavones appear to exhibit differential effects on inflammatory processes. For example, IFN-γ induced pSTAT1 was reduced in Caco-2 cells (human epithelial colorectal adenocarcinoma) upon treatment with genistein^5^. Similarly, other studies have shown that LPS-induced STAT1 activation is abrogated when microglial cells were cultured with genistein or equol^6^. These data are consistent with reports indicating that soy isoflavones can downregulate inflammatory cytokine production (IL-6, IL-8, TNF-α, IL-12) in several different immune cell subtypes^7^. Others have shown that phytoestrogens (genistein and daidzein) are weakly estrogenic and can activate natural killer (NK) cell activity at concentrations of 0.1 to 10 µM *in vitro*^8,9^. Thus, it is clear that soy isoflavones and their metabolites have modulatory effects on inflammatory signaling processes. However, the differential effects of individual soy isoflavones and their host or microbial metabolites on NK cell activity, one component of a complex immune response, is still poorly understood.

Understanding the role of isoflavones and their metabolites in regulating NK cell activity is critical due to the importance for NK cells as mediators of cytokine secretion and inflammatory responses including infection, tissue damage and immunosurveillance against tumors^10^. These actions are accomplished not only by the well-characterized cytotoxic effects of NK cells, but also by their ability to produce IFN-γ in response to inflammatory cytokine stimuli such as IL-12 and/or IL-18^11,12^. Together these cytokines shape the immune response in a manner that orchestrates inflammatory changes that regulate control of cancers and pathogens.

We hypothesized that soy isoflavones and their metabolites represent dietary components that impact NK cell mediated immune function. In this report, we describe how two soy isoflavones (genistein and daidzein) and two microbial metabolites (O-DMA and equol) impact NK cell activity. We found that genistein and equol were potent inhibitors of IL-12/IL-18 induced NK cell IFN-γ production, but had no effect on NK cell cytotoxicity against target cells. Additionally, treatment of NK cells with genistein reduced IL-12/IL-18 induced phosphorylation of ERK and total phosphorylated-tyrosine. Genistein also reduced the IL-12/IL-18 induced surface expression of IL-18 receptor-alpha (IL-18 Rα) by CD56^+^ NK cells, but had no effect on IL-12 receptor-beta-1 (IL-12Rβ1) expression. Further, murine experiments showed that mice fed a soy-enriched diet produced lower plasma IFN-γ levels following *in vivo* challenge with IL-12 and IL-18. Together our data provide novel evidence for soy phytochemicals as modulators of cytokine communication involving NK cells *in vitro* and *in vivo*. Importantly, these data suggest that exposure to soy isoflavone metabolites may impact the ultimate biologic response to a soy based dietary intervention. Demonstrating that dietary components impact NK cell mediated immune responses has implications for future clinical application.

## Results

### Soy isoflavones and their metabolites do not affect immune cell viability

**Figure 1:**
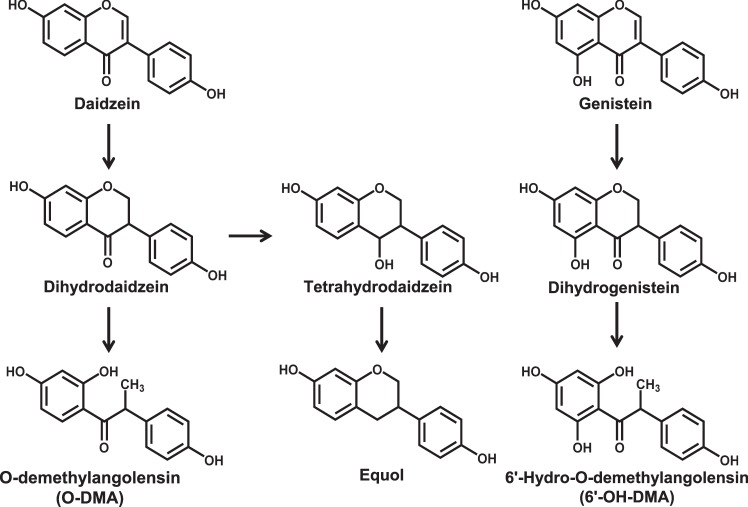
Soy isoflavones and their metabolite compounds. Genistein and daidzein are two of the most abundant isoflavones found in soy. Daidzein can be further metabolized into secondary compounds, O-demthylangolensin (O-DMA) and Equol.

**Figure 2:**
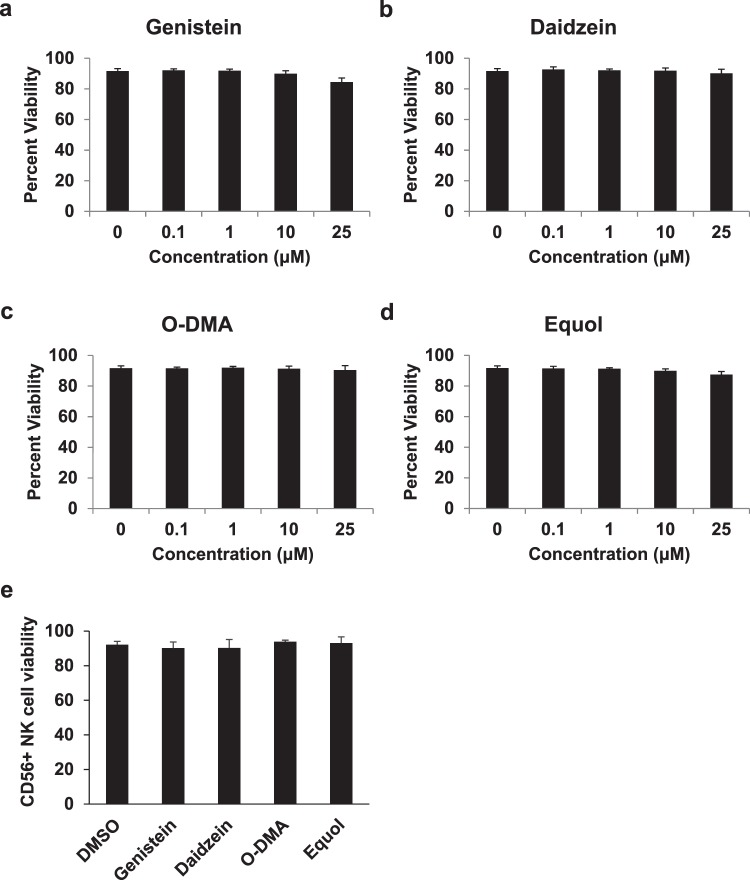
Soy isoflavones and their metabolites do not affect immune cell viability. Healthy human donor PBMCs (**a**–**d**) and CD56^+^ NK cells (**e**) were incubated for 72 hours with genistein, daidzein, O-DMA, and equol. Cells were then assessed for viability.

Daidzein and genistein represent two prominent isoflavones present in soy that are metabolized by the intestinal microbiota into unique secondary metabolites such as equol and O-DMA (Fig. 1)^13–15^. *In vitro* studies first examined whether these particular compounds impact immune cell viability. At physiological and pharmacologic concentrations (25 µM) no decreases in viability of PBMCs (Fig. 2a–d) or CD56^+^ enriched NK cells (Fig. 2e, Supplemental Fig. 1) were observed following a 3-day incubation with the soy isoflavones (genistein and daidzein) or metabolites (Equol and O-DMA).

### Genistein and Equol inhibit IL-12/IL-18-induced IFN-γ production

**Figure 3:**
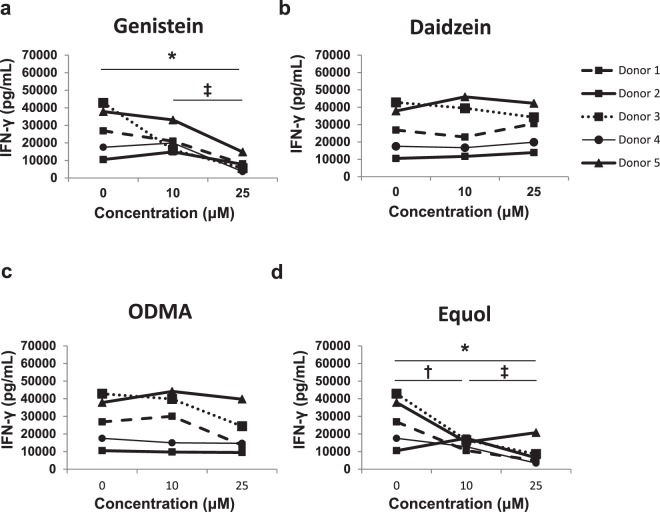
Genistein and equol abrogate IL-12/IL-18 induced IFN-γ production by human primary PBMCs. Human primary PBMCs were cultured with (**a**) genistein, (**b**) daidzein, (**c**) O-DMA, and (**d**) equol for 4 hours and then stimulated with 20 ng/ml IL-12 and 50 ng/ml IL-18 for 72 hours. Culture supernatants were tested for IFN-γ by ELISA. Data is representative from 5 healthy donors. Means +/− STD. *p < 0.05, 25 μM concentration compared to DMSO; ^†^p < 0.05, 10 µM concentration compared to DMSO; ^‡^p < 0.05, 25 µM concentration compared to 10 µM.

We next examined whether soy isoflavones or metabolites could alter the *in vitro* response of immune cells to canonical inflammatory stimuli. For these studies, the response to IL-12/IL-18 was first examined, as these cytokines are critical orchestrators of immune function and represent a potent inflammatory stimulus. PBMCs were cultured with varying concentrations of soy isoflavones and metabolites for 4 hours and then stimulated for 72 hours with IL-12/IL-18. PBMCs remained incubated with soy isoflavones/metabolites for the duration of IL-12/IL-18 stimulation. A significant decrease in IFN-γ production in cell culture supernatants from PBMCs treated with genistein (Fig. 3a; p = 0.0023) or equol (Fig. 3d; p = 0.006) was observed. In contrast, culture of PBMCs with daidzein (Fig. 3b) or O-DMA (Fig. 3c; p = 0.1020) did not significantly reduce IFN-γ production *in vitro*.

### Genistein and Equol decrease IL-12/IL-18-induced IFN-γ production by NK cells

**Figure 4:**
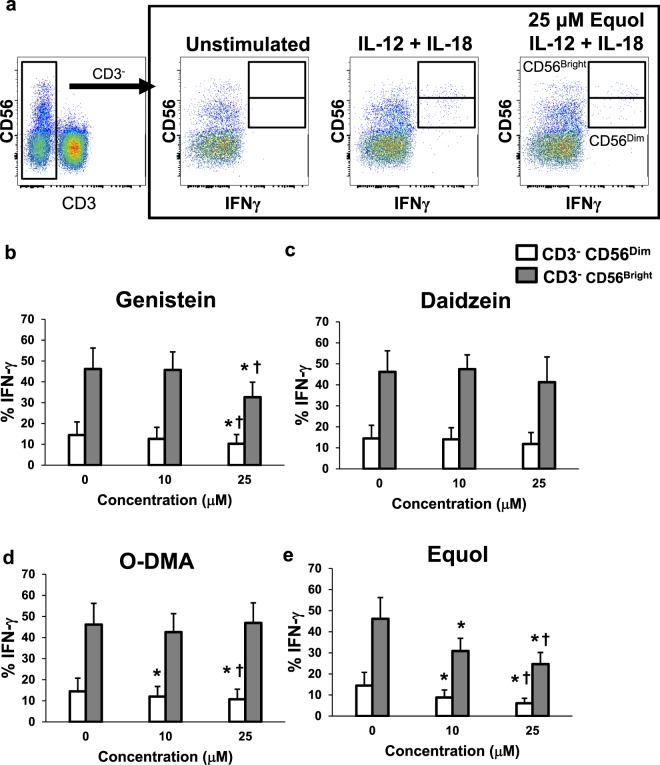
Genistein and Equol decrease IL-12/IL-18 induced NK cell IFN-γ production. Human primary PBMCs were cultured with soy compounds for 4 hours and then stimulated with 20 ng/ml IL-12 and 50 ng/ml IL-18 for 72 hours. (**a**) Cells were initially gated based on forward and side scatter to exclude debris, then cells were gated based on CD3 and CD56 expression as NK cells (CD3^−^CD56^Bright^ NK cells, CD3-CD56^Dim^ NK cells, or CD3^+^CD56^+^NKT cells based on evident positive and negative cell populations. Intracellular IFN-γ was then assessed in these populations via intracellular flow cytometry. The positive gate was set based on the level of fluorescence observed in unstimulated cells, which coincided with the level of fluorescence observed in unstained control cells (not pictured). (**b**) IFN-γ intracellular staining in CD3^−^NK^Dim^ (open) and CD3^−^CD56^Bright^ (shaded) NK cells. Cells were pre-treated prior to IL-12/IL-18 stimulus in DMSO (0 μM), (**b**) genistein, (**c**) daidzein, (**d**) O-DMA or (**e**) equol. Data is representative from 3 healthy donors. Means +/− STD. *p < 0.05, concentration compared to DMSO; ^†^p < 0.05, 25 µM concentration compared to 10 µM.

NK cells are a predominant cell type activated in response to stimulation with IL-12 and IL-18, although differentiated T lymphocytes and NKT cells can also be impacted to a lesser degree^16,17^. To determine the impact of soy phytochemicals on IFN-γ production from each of these immune cell subsets, bulk PBMCs were cultured with soy isoflavones or their metabolites for 4 hours prior to a 24 hour stimulation with IL-12/IL-18. Cells were then stained for intracellular IFN-γ via flow cytometry. We found that CD3^−^CD56^Bright^ NK cells were the primary producer of IL-12/IL-18 induced IFN-γ (Fig. 4), which significantly decreased when incubated with genistein (Fig. 4b; p = 0.0147) or equol (Fig. 4c; p < 0.0001). We also observed similar decreases of intracellular IFN-γ by CD3^−^CD56^dim^ NK cells when treated with genistein (p = 0.0153) and equol, (p = 0.0003) however overall percentages of positive cells were lower compared to CD3^−^CD56^Bright^ NK cells (Fig. 4b). Similar to data obtained with cell culture supernatants, incubation of CD3^−^CD56^dim^ NK cells with ODMA also reduced intracellular IFN-γ compared to DMSO control (Fig. 4b; p = 0.0078). Percentages of IFN-γ^+^ CD3^−^CD56^Bright^ or CD3^−^CD56^dim^ were not affected when incubated with daidzein (Fig. 4c). Intracellular staining also revealed a decrease in IFN-γ from CD3^+^CD56^+^ NKT cells when cultured with genistein and equol (Supplemental Fig. 2). The overall frequency of IFN-γ producing NKT cells was much lower than NK cells, however, this trend in response to genistein and equol was consistent across multiple donors. Further, the overall IFN-γ production by CD3^+^ T cells and CD14^+^ monocytes was considerably lower than NK cells, however not appreciably impacted by the soy isoflavones or metabolites.

### Soy isoflavones and NK cytotoxicity

**Figure 5:**
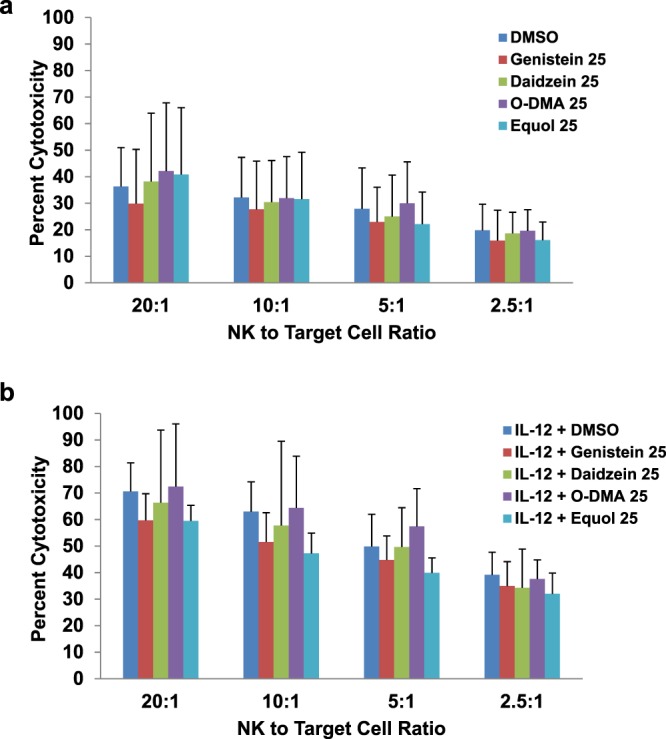
Soy compounds do not affect NK cell cytotoxicity. Human primary NK cells were isolated from healthy donor PBMCs and were cultured with 25 µM of soy compounds (Genistein, Daidzein, O-DMA, Equol) for 4 hours then (**a**) unstimulated or (**b**) stimulated with IL-12 (10 ng/ml) overnight. NK cells were co-cultured with ^51^Cr labeled target cells (K562) for 4 hours and chromium release was analyzed to determine percent cytotoxicity. Data is representative from 4 healthy donors.

Another critical function of NK cells is their ability to elicit cytotoxic activity against target cells^10,18,19^. This cytotoxic function of NK cells can be enhanced in the presence of cytokines such as IL-12^20^. We tested whether soy isoflavones or their metabolites alter NK cytotoxicity. For these experiments, CD56^+^ NK cells negatively selected from healthy normal donors were pre-incubated for 4 hours with 25 µM of the soy compounds, and then stimulated with and without IL-12 overnight (Fig. 5a,b). Following stimulation, NK cells were co-cultured with Cr^51^ labeled K562 target cells for 4 hours. As expected, IL-12 enhanced NK cytotoxicity against K562 target cells. However no consistent difference in cell killing was observed across all effector:target ratios when cells were pre-incubated with soy isoflavones or metabolites. For instance, in unstimulated NK cells, only three individual comparisons (equol vs. DMSO at a ratio of 5:1, genistein vs. DMSO at ratios of 20:1 and 2.5:1) were below the 0.0625 significance level, where only one would be expected by chance. For NK cells stimulated with IL-12, only one comparison (equol vs. DMSO at a ratio of 10:1) was below the threshold, the same as would be expected by chance.

### Selective modulation of cytokine-induced signal transduction events by genistein

**Figure 6:**
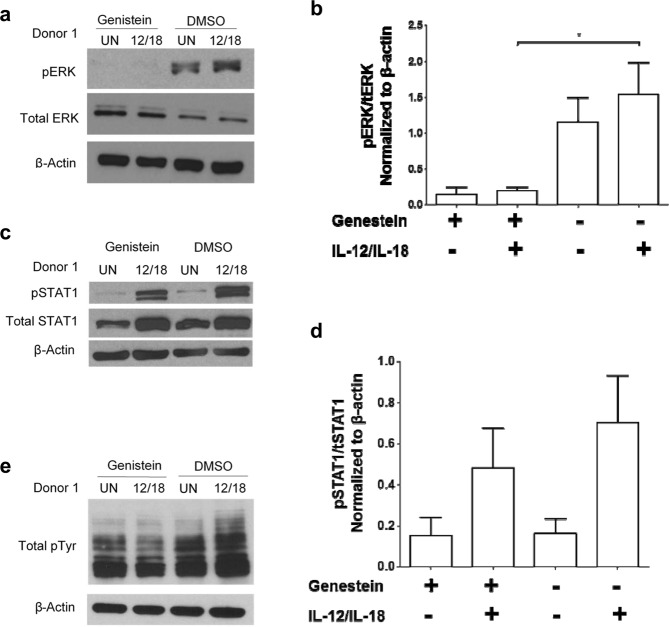
Genistein modulates cellular signaling events. PBMC from healthy donors were cultured with 25 µM of genistein or DMSO (vehicle control) for 4 hours then stimulated with 20 ng/ml IL-12 and 50 ng/ml IL-18 for either 24 hours of additional incubation (long-term), or 15 minutes of additional incubation (short-term) to analyze canonical signal transduction events. (**a**) Immunoblot analysis of pERK was conducted in short-term lysates to focus on signaling events proximal to initial IL-18 receptor engagement. (**b**) Densitometry analysis of western blot pERK levels across conditions normalized to total ERK and β-actin levels. (**c**) Immunoblot analysis of pSTAT1 was conducted in long-term lysates where IFN-γ, an upstream STAT1 activating stimulus was at high levels (**d**) Densitometry analysis of western blot phosphor-STAT1 levels across conditions normalized to total STAT1 and β-actin levels. (**e**) Immunoblot analysis of pTyr was conducted in long-term lysates where IFN-γ and pan-pTyr activation was at high levels. Data are presented from three separate donors with similar results. In two of three experiments, total STAT1 or ERK, along with β-actin are included as controls and were run using identical lysates on separate gels, given similar molecular weight of some proteins. In one of three experiments, each phospho-specific Ab blot was stripped and re-probed for detection of loading controls. (Veh = vehicle; 12/18 = IL = 12 + IL-18 stimulation) Immunoblots were cropped for presentation purposes and original blots are provided as Supplemental Fig. 4.

The impact of genistein on canonical cytokine-induced phosphorylation of STAT1 (pSTAT1), ERK (pERK), and total tyrosine (pTyr) was next examined in cells pre-treated with genistein and exposed to IL-12/IL-18 stimulation. These signaling proteins are of interest given the abundance of IFN-γ produced in response to IL-12/IL-18, and the known role of ERK in signaling downstream of the IL-18R^21,22^. The phosphorylation of ERK (Fig. 6a,b, Supplemental Fig. 3) and total tyrosine (Fig. 6e, Supplemental Fig. 3) was abrogated by a 4 hour genistein pre-treatment in both untreated PBMCs and those exposed to IL-12/IL-18 stimulation as compared to controls. Similar trends were observed for STAT1 phosphorylation although these data did not reach statistical significance (Fig. 6c,d).

### Genistein decreases expression of IL-18Rα on NK cells

**Figure 7:**
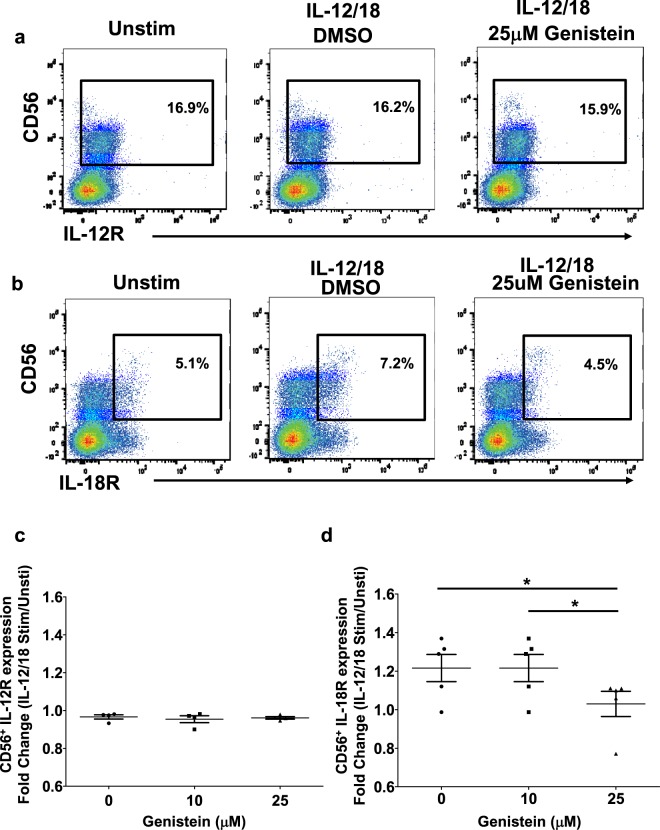
Genistein reduces expression of IL-12/18 induced expression of IL-18Rα. PBMCs from healthy human donors (n = 5) were cultured with 10 or 25 µM of genistein or DMSO (vehicle control) for 4 hours then stimulated with 20 ng/ml IL-12 and 50 ng/ml IL-18 for either 24 hours of additional incubation. IL-12 receptor (IL-12Rβ1) and IL-18 receptor (IL-18Rα) surface expression was analyzed by flow cytometry. Representative dot plots of CD56^+^ NK cells expressing (**a**) IL-12Rβ1 and (**b**) IL-18Rα. (**c**) IL-12Rβ1 or (**d**) IL-18Rα expression from 5 different donors was analyzed by fold change of unstimulated over IL-12/18 stimulated NK cells. Means +/− STD. *p < 0.01, concentration compared to DMSO control (0 μM).

Given the ability of genistein to limit cytokine-induced signaling via ERK, we were interested in examining the expression of IL-12 and IL-18 receptors on NK cells. Healthy donor PBMCs were pre-incubated with genistein, stimulated with IL-12/18, and analyzed for expression of IL-12 and IL-18 receptor expression on CD56^+^ NK cells (Fig. 7a,b). Genistein pre-treatment did not impact expression of IL-12Rβ1 on CD56^+^ NK cells (Fig. 7c). However, genistein reduced the expression of IL-18Rα on CD56^+^ NK cells stimulated with IL-12/18 compared to unstimulated cells (Fig. 7d; p < 0.01).

### Soy-enriched diet reduces IL-12/18 induced IFN-γ responses *in vivo*

**Figure 8:**
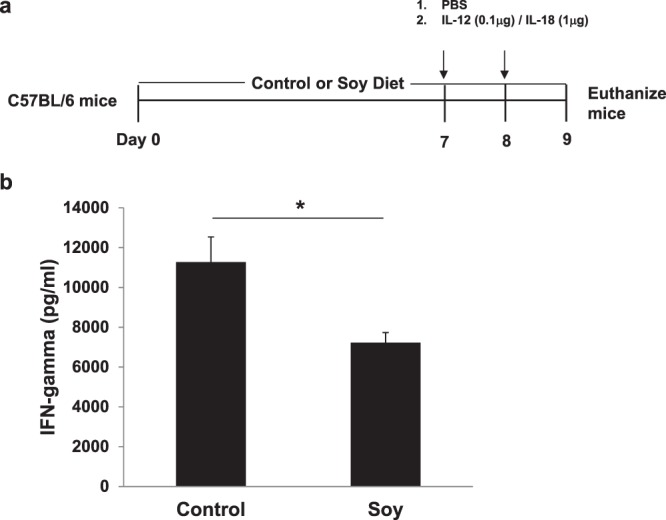
Soy-enriched diet inhibits IL-12/IL-18 induced IFN-γ responses *in vivo*. C57BL/6 mice were (**a**) administered a control or a 0.3% soy-enriched diet for 7 days and then intraperitoneally injected with 0.1ug IL-12 and 1ug IL-18 on days 7 and 8. (**b**) On day 9 mice were euthanized and plasma was assessed by ELISA for IFN-γ levels. Means +/− STD, *p < 0.0001, soy fed mice compared to control fed mice.

To better approximate the impact of physiologically relevant soy phytochemical concentrations consumed in the diet, we modeled the potent inflammatory stimulus of IL-12 and IL-18 *in vivo*. Previously published work from our group reported that C57BL/6 mice injected with the combination of IL-12 and IL-18 resulted in IFN-γ from primarily only the NK cell population^21^. For these studies, C57BL/6 mice were fed a control or soy-isoflavone enriched diet for one week and injected intraperitoneally with IL-12/IL-18 (Fig. 8a). Plasma levels of IFN-γ were significantly reduced in mice fed a soy-enriched diet compared to the control diet (Fig. 8b; p < 0.0001).

## Discussion

Individual soy isoflavones and their microbial metabolites may have distinct effects on immune function. The present study focuses on how these compounds impact the diverse biologic functions of NK cells. In particular, we demonstrate that the soy isoflavone, genistein and the daidzein metabolite, equol are particularly adept at abrogating IL-12/IL-18 induced production of IFN-γ by NK cells. A previous report by Nishio *et al*. indicate higher concentrations of genistein (10 µg/mL) can decrease cytotoxicity of IL-2 stimulated NK cells^23^. In contrast, these soy isoflavones or their metabolites did not alter cell viability or the cytotoxic function of NK cells either with or without IL-12 stimulation. Concentrations used in our study were lower (25 µM), and this prior study was limited to only genistein in the context of IL-2 stimulation, potentially accounting for the different observations related to cytotoxic activity. Importantly, the ability of soy components to reduce IL-12/IL-18 induced IFN-γ was recapitulated in mice receiving a soy-enriched diet, highlighting the physiological relevance of these observations. Together these results indicate the need for further mechanistic study on how individual soy components or metabolites derived from the host or microbiota can modulate the function of specific cellular components of the immune system. This data has important implications for understanding how NK cells may regulate chronic and acute inflammatory responses^10,18,19^.

Our motivation to examine how individual soy components or metabolites impact immune function stems in part from results of a prior human clinical study^4^. We observed that men with prostate cancer received a dietary soy intervention that resulted in down-regulation of several pro-inflammatory cytokines in the plasma^4^. A particularly informative companion study using blood and urine from these same men was also conducted before and after intervention^2^. This pharmacokinetic study demonstrated that men clustered into four distinct soy metabolite signatures based on their ability to metabolize daidzein. We postulated that this observation may open further lines of investigation as to how the proportions of isoflavones or their metabolites can alter inflammation and the immune response.

There is currently a gap in our understanding as to how soy components impact specific facets of the immune response, and few studies have systematically explored the complexity of isoflavone metabolites as they relate to immune function. For example, *in vitro* studies investigating soy isoflavones and immune function have focused on the parent isoflavones, genistein and daidzein. Past studies have investigated the effects of genistein on IFN-γ responses, however these studies have utilized stimuli such as PMA/Ionomycicn, ConA, or PHA to induce a response^24–26^. These stimuli are of more limited physiologic relevance and these studies were focused primarily on genistein rather than other soy phytochemicals. Indeed, our prior clinical studies with dietary soy intervention^2,4^ and others have more recently appreciated the complexity of soy isoflavone metabolism with the need to expand our investigation of isoflavone metabolite byproducts, O-DMA and equol. In clinical trials of patients with prostate cancer receiving a soy bread intervention, detectable genistein, daidzein, O-DMA and equol were noted in both plasma and urine from patients^2^. Although beyond the scope of the current study, it is important to note that understanding the host and microbial metabolism of soy isoflavones into multiple metabolites with local or systemic bioactivity is only beginning to emerge. In support of a role for microbial regulation of isoflavone metabolism are studies completed in rats lacking soy metabolizing gut microbiota. These animals were unable to metabolize isoflavones unless inoculated with bacterial strains known to metabolize isoflavones^27^. Taken together, these data suggest the microbiome is a relevant mediator of isoflavone metabolism. Thus, there is an emerging opportunity to delineate the multi-directional communication between diet, microbiota and immune response and its impact upon inflammation or disease^2,28,29^.

In theory, the ability of different populations and patients to metabolize soy isoflavones could also influence the potential outcomes and effect on immune interventions targeting inflammatory conditions. For example, a percentage of individuals effectively metabolize the soy isoflavone daidzein into equol more efficiently^3^, which could lead to a more drastic reduction in NK cell IFN-γ production compared to non-metabolizers. Thus, the individual ability to metabolize isoflavones could potentially lead to differential effects on immune responses. This emphasizes the importance and novelty of this report as we observed not only the effect of soy isoflavones on NK cell function but also their metabolite byproduct equol. The monitoring of isoflavone metabolism in clinical settings will be informative when administering dietary soy with the intent of health benefit.

Several questions remain when considering the activity of soy on immune response, and in particular, its actions on NK cell biology as demonstrated in this study. It will be important in future studies to determine whether these actions of soy on NK cells may be beneficial or detrimental to immune responses in the settings of infection or cancer. Indeed, NK cells are key mediators of immune surveillance against virally-infected cells or developing tumors, and in communicating with adaptive immune cells by virtue of their cytokine production^10^. Thus, depending on the context, soy effects on NK cell activity might have unpredictable effects on these disease processes or the response to vaccines or other immune modulatory therapies. Additionally, we reported that genistein can reduce the expression of IL-18Rα on CD56^+^ NK cells and phosphorylated tyrosine protein levels. The ability of soy isoflavones to reduce IL-18 receptor signaling could be a possible mechanism for dampened IFN-γ production by NK cells, although the robust changes in multiple cellular signaling pathways imply there are likely pleiotropic effects mediated by soy that culminate in global reductions in cytokine production. Together these results allow for a greater understanding of the complexity of isoflavone metabolites and their impact upon NK cell biology.

## Methods

### Cell culture and Reagents

All cells were cultured in RPMI-1640 (Gibco, Grand Island, NY) with 5% FBS, 1% L-Glutamine and antibiotic/antimycotic (Gibco). Soy isoflavones, genistein and daidzein, were purchased from LC Laboratories with greater than 99% purity (Woburn, MA). Equol was purchased from Toronto Research Chemicals (Toronto, ON). O-demtheylangolensin (O-DMA) was purchased from Planteck UK (Berkshire, UK). The control diet was the AIN-93 G (Table 1). A soy-enriched irradiated diet was prepared by Envigo (Madison, WI) consisting of the AIN-93G diet with 7.246 g of soy extract per 1 kg of diet substituting 7% corn oil, for soybean oil, in the AIN-93G diet formulation. The AIN-93G diet with 7% corn oil (in lieu of soybean oil) was used as a base for both the soy-enriched and vehicle control diets. The soy extract (Solgen 40 from Solbar, Ashdod, Israel) is a 40.25% soy isoflavone (w/w) soy bean extract, and when incorporated results in approximately 2.92 g/isoflavones per 1 kg of diet. Recombinant human and murine IL-12 was purchased from Peprotech, Inc. (Rocky Hill, NJ). Recombinant human and murine IL-18 was purchased from R&D Systems Inc.

**Table 1:** Composition of soy-enriched diet for *in vivo* mouse studies.

Composition of Experimental Diets		
Grams/100 g Total Diet		
Ingredients	Control Diet	Soy Diet
Casein	20.0	20.0
L-Cystine	0.3	0.3
Corn Starch	39.2234	38.4988
Maltodextrin	13.2	13.2
Sucrose	10.0	10.0
Corn Oil	7.0	7.0
Fiber [Non-nutritive cellulose]	5.0	5.0
Mineral Mix *[AIN-93G-MX formulation]*	3.5	3.5
Vitamin Mix *[AIN-93G-VX formulation]*	1.5	1.5
Choline Bitartrate	0.275	0.275
Vitamin K1, phylloquinone	0.0002	0.0002
TBHQ, antioxidant	0.0014	0.0014
Soy Isoflavone Extract *[Solbar Hatzor Ltd. Solgen 40]*	0.0	0.7246
***(mg/100*** ***g diet)***		
Total Isoflavones	0.0	291.65
Genistein/Genistin	0.0	167.12
Soy Protein	0.0	72.39

0.3% soy isoflavone-enriched, irradiated AIN-93G diet (Soy Diet). 7.246 g of a 40% soy isoflavone (w/w) soy bean extract (Solgen 40 from Solbar, Ashdod, Israel) was incorporated per 1 kg total diet, substituting 7% corn oil, for soybean oil, in the AIN-93G diet formulation. The AIN-93G diet with 7% corn oil (in lieu of soybean oil) was used as a vehicle control (Control Diet).

### Isolation and differentiation of human blood cells

Peripheral blood mononuclear cells (PBMCs) were isolated from source leukocytes of healthy adult donors (American Red Cross, Columbus, OH) with Ficoll-Paque (Amersham, Uppsala, Sweden) as previously described^30,31^. For NK cells, CD56^+^ cells were isolated from human donor blood by negative selection RosetteSep antibody cocktail (STEMCELL Technologies, Vancouver, BC).

### Analysis of IFN-γ by ELISA

PBMCs from multiple healthy human donors were pre-incubated with DMSO vehicle control or isoflavones/metabolites for 4 hours, and were subsequently stimulated with 20 ng/ml of recombinant human IL-12 and 50 ng/ml of IL-18 for 72 hours. Cell culture supernatants were analyzed for the presence of IFN-γ. PBMCs remained incubated with soy isoflavones/metabolites for the duration of IL-12/IL-18 stimulation. Samples were run for IFN-γ using commercial ELISA kits (R & D Systems, Inc.) in duplicate per manufacturer’s recommendations.

### Flow cytometric analysis

PBMCs were stimulated with 20 ng/ml of IL-12 and 50 ng/ml of IL-18 for 18 hours. Cells were incubated with Golgi-stop (BD Biosciences) for 4 hours and different immune cell populations were assessed for intracellular IFN-γ (BD Biosciences) production. Expression of IL-12Rβ1 and IL-18Rα were measured as extracellular phenotypic markers by incubating samples for 1 hour on ice, washing and storage at 4 °C until analysis. Specific antibodies included CD3-AlexaFluor 488, CD56-AlexaFluor 656, CD11c-APC, CD14-AlexaFluor 488, CD66b-AlexaFluor 647, IL-18Rα (clone H44) and IFN-γ-PE (BD Biosciences). Anti-IL-12Rβ1 (clone REA242) was purchased from Miltenyi Biotech. Appropriate isotype control antibodies for each fluorochrome were used as negative controls. All samples were run on a BD LSR II flow cytometer, and analyzed with FlowJo (Tree Star, Inc.).

### NK cytotoxicity assay

CD56^+^ NK cells isolated from normal human donors by negative selection (RosetteSep antibody cocktail (STEMCELL Technologies) were plated in 96-well V-bottomed plates treated with or without IL-12 (10 ng/ml) overnight in RPMI-1640 supplemented with 10% human AB serum media at 37 °C. Eighteen hours later, ^51^Cr-labeled K562 tumor cells were incubated with NK cells at various effector:target (E:T) ratios. Following a 4-hour incubation, supernatants were harvested and chromium release was assayed and percent lysis was calculated as previously described^32^. Briefly, spontaneous release represents ^51^Cr release from target cells in medium alone (minimum value), and maximum release is ^51^Cr release from target cells in medium + 10% SDS. The NK cells were plated at a concentration of 1 × 10^6^/ml to obtain an initial ratio of 20:1, followed by a serial dilution for the other E:T ratios. The calculation for the percent lysis is an average of samples plated in triplicate, [(Raw Gamma Count-Average Minimum Value)/Average Maximum Value]*100.

### *In vivo* IL-12/18 treatments

All murine experiments were conducted under an Institutional Animal Care and Use Committee (IACUC) protocol (2009A0178) approved by the IACUC committee at The Ohio State University (Columbus, OH) in compliance with their Animal Care and Use Program. C57BL/6 J mice (Stock No: 000664; The Jackson Laboratory, Bar Harbor, ME) 6–8 weeks of age, were administered the AIN-93G-based control diet or an AIN-93G-based 0.3% soy-enriched diet (Envigo) for 7 days. Mice were then injected with 0.1 µg of recombinant murine IL-12 (R&D Systems) and 1 µg IL-18 (R&D Systems) or PBS per mouse on days 7 and 8^33,34^. Mice were euthanized after 48 hours (Day 9) of exogenous cytokine injections to test for plasma IFN-γ concentrations.

### Immunoblot Analysis

Western blot analysis was completed on cell lysates from normal human donor PBMCs pre-incubated with isoflavones or metabolites for 4 hours and stimulated with IL-12/IL-18 (20 ng/ml, 50 ng/ml, respectively) for 24 hours (long-term) or 15 minutes (short-term). Immunoblots were probed with antibodies specific for pSTAT1 (catalog #9171 L), pERK (catalog #4377 S), ERK (catalog #4695 S), all purchased from Cell Signaling Technology^35,36^ and STAT1 (catalog #610115) purchased from BD Biosciences. Primary antibody to detect total phosphorylated tyrosine (clone PY99, catalog #sc-7020) was purchased from Santa Cruz Biotechnology^37^. Following incubation with appropriate horseradish peroxidase-conjugated secondary antibodies, immune complexes were detected using the SuperSignal West Pico Chemiluminescent Substrate (Thermo Scientific/Pierce, Rockford, IL). β-actin (4967 S; Cell Signaling Technologies) was used as a loading control. Densitometry was performed using ImageJ software. All densitometric calculations are derived from n = 2 normal donors.

### Statistics

Mixed-effects models were used to analyze data from donor cell experiments, including a random effect for donor and fixed effects for the doses of soy isoflavones. The Tukey-Kramer method was used to adjust for multiple comparisons. A two-sample t-test was used to compare IFN-γ concentrations from the diet enrichment experiment. Data were log-transformed prior to analysis to improve normality and stabilize the variance. Comparisons in the NK cytotoxicity experiments used α = 0.0625 (1/16) to set the expected number of false positives to one following the method of Gordon *et al*.^38^. All analyses were conducted in SAS v9.4 (SAS Institute, Cary, NC).

## Supplementary information

Supplemental Data All

## Data Availability

The datasets generated during or analyzed during the current study are available from the corresponding author on reasonable request.
