# Supplementary material for: Soy isoflavones and their metabolites modulate cytokine-induced natural killer cell function
Source: Sci Rep. 2019 Mar 25;9:5068. doi: 10.1038/s41598-019-41687-z (PMC6433892; doi:10.1038/s41598-019-41687-z)

Soy isoflavones and their metabolites modulate cytokine-induced natural killer cell function.

Thomas A. Mace[1,2], Michael B. Ware[8], Samantha A. King[2], Shannon Loftus[2], Matthew R. Farren[8], Elizabeth McMichael[4,5], Steven Scoville[6], Connor Geraghty[2,3], Gregory Young[7], William E. Carson, III[4,5], Steven K. Clinton[2,3], and Gregory B. Lesinski[8]

[1]Division of Gastroenterology Hepatology Nutrition, [2]Department of Internal Medicine, [3]Division of Medical Oncology, [4]Division of Surgical Oncology, [5]Department of Surgery, [6]Biomedical Sciences Graduate Program, Medical Scientist Training Program, [7]Center for Biostatistics, The Arthur G. James Cancer Hospital and Richard J. Solove Research Institute, Comprehensive Cancer Center, The Ohio State University, Columbus, OH, USA. [8]Department of Hematology and Medical Oncology, Winship Cancer Institute of Emory University.

(Supplementary Data Files)

Supplemental Figure 1

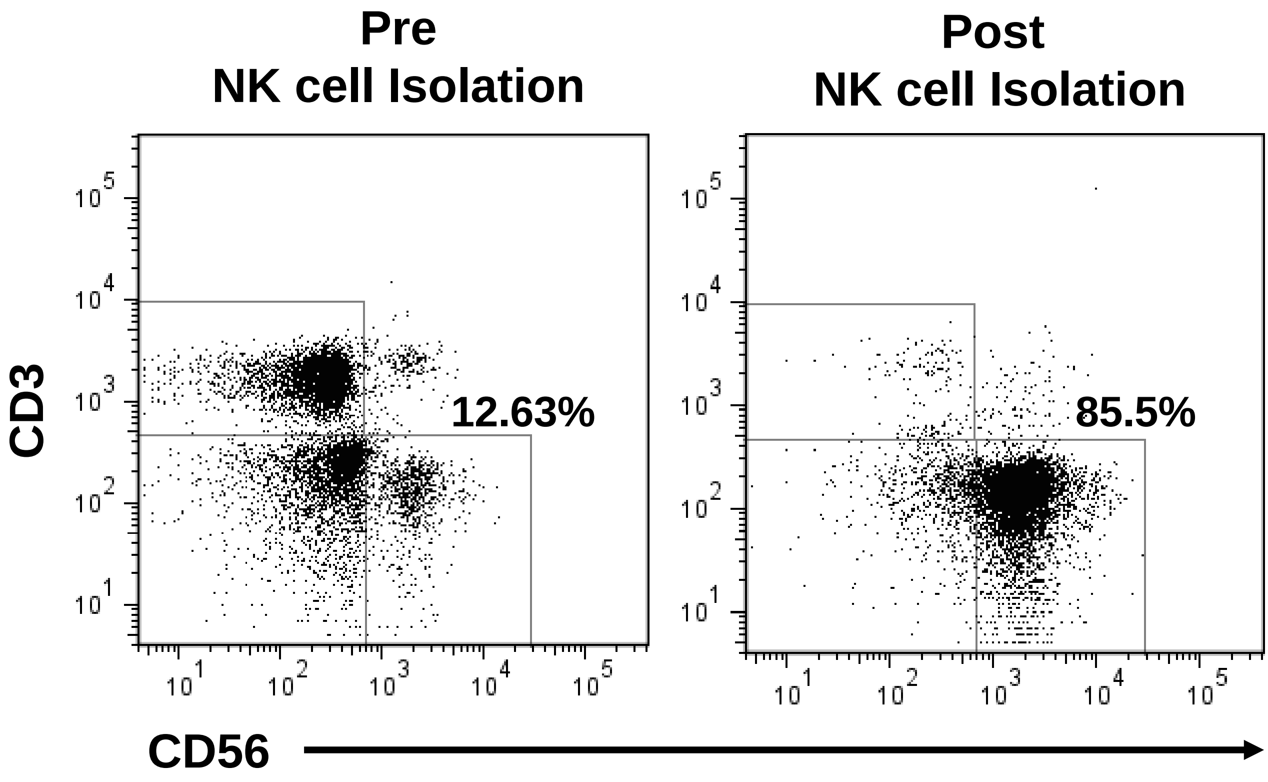

Supplemental Figure 1. Representative NK cell purity from healthy donor PMBC isolation. Human primary PBMC were negatively selected for $CD56^{+}CD3^{-}$ NK cells using Rossettesep antibody cocktail. Cell were stained by flow cytometry pre- and post-isolation to determine $CD56^{+}CD3^{-}$ positivity.

Supplemental Figure 2

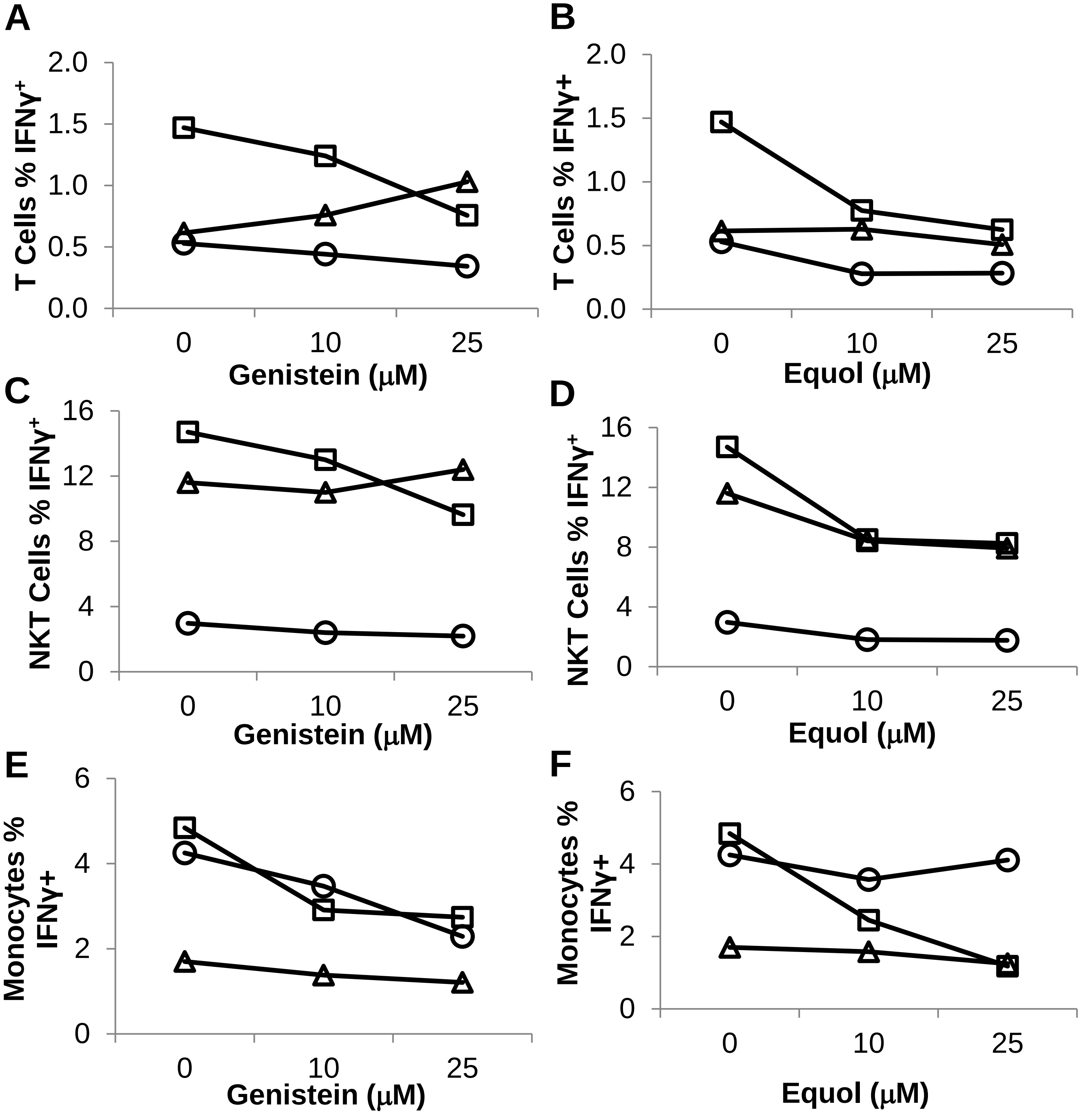

Supplemental Figure 2: Effect of genistein and Equol on IL-12/IL-18 induced IFN-γ production by T and NKT cells. Human primary PBMC were cultured with soy compounds for 4 hours and then stimulated with 20ng/ml IL-12 and 50ng/ml IL-18 for 72 hours. Cells were gated into T, NKT or monocyte cell populations as described in Fig. 4. IFN-γ production from stimulated T cells treated with A) genistein or B) equol. IFN-γ production by equol treated Tcells. IFN-γ production from stimulated NKT cells treated with C) genistein or D) equol. IFN-γ production from stimulated monocytes treated with E) genistein or F) equol. Cells were pretreated prior to IL-12/IL-18 stimulus in DMSO (black bars). Data is representative of 3 healthy donors. Means +/- STD.

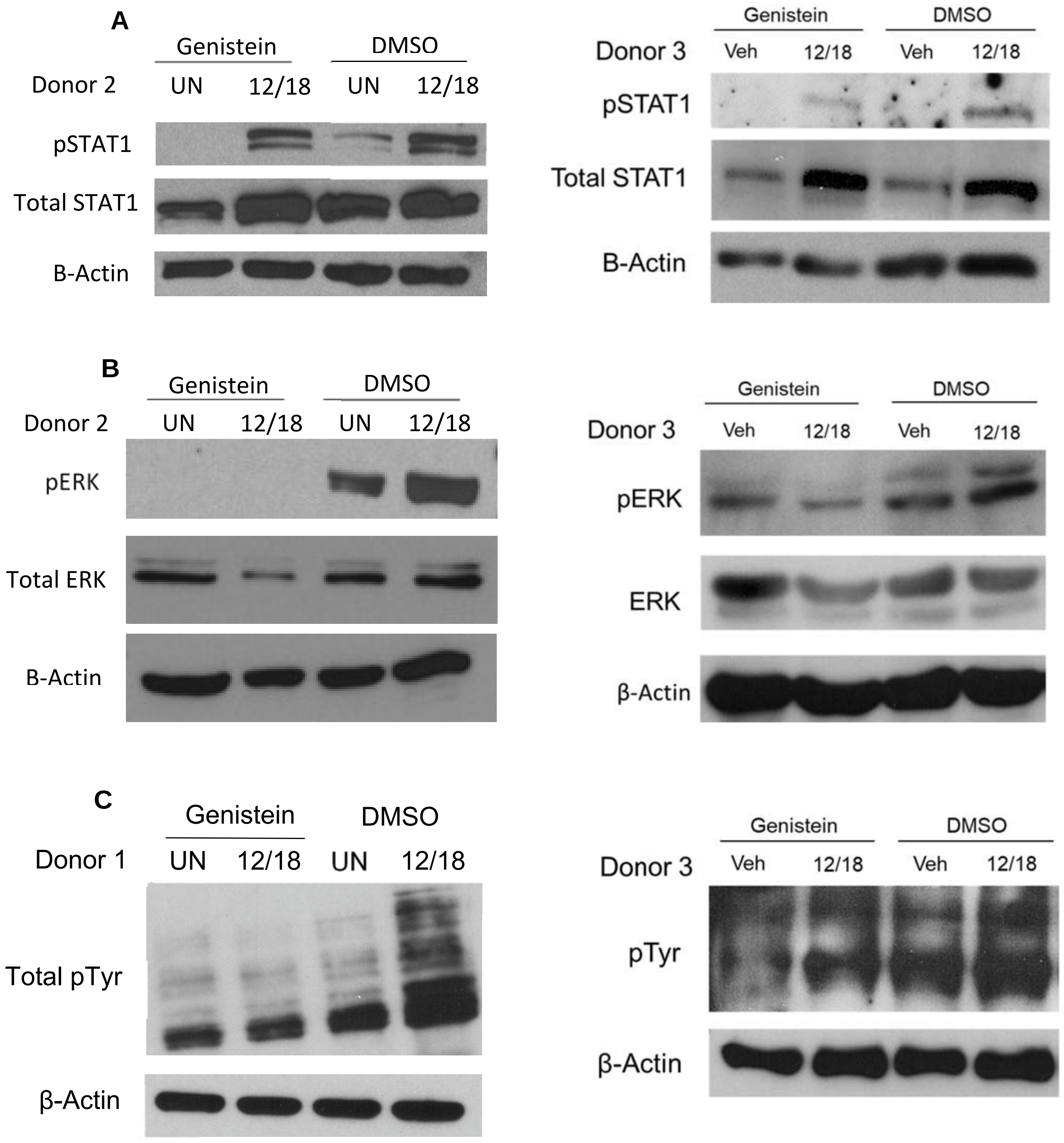

Supplemental Figure 3. Immunoblot images of additional donors included in the densitometry analysis shown in figure 6. (**A**) Immunoblot analysis of pSTAT1 activation after 24hrs of IL-12/18 stimulation in the presence of absence of Genistein for Donor 2 and Donor 3 (**B)** Immunoblot analysis of pERK activation after 1hr of IL-12/18 stimulation in the presence or absence of Genistein for Donor 2 and Donor 3 (**C)** Immunoblot analysis of pTyr activation after 24hr of IL-12/18 stimulation in the presence or absence of Genistein for Donor 2 and Donor 3.

Supplemental Figure. 4. Uncropped Western Blots for Figure 6.

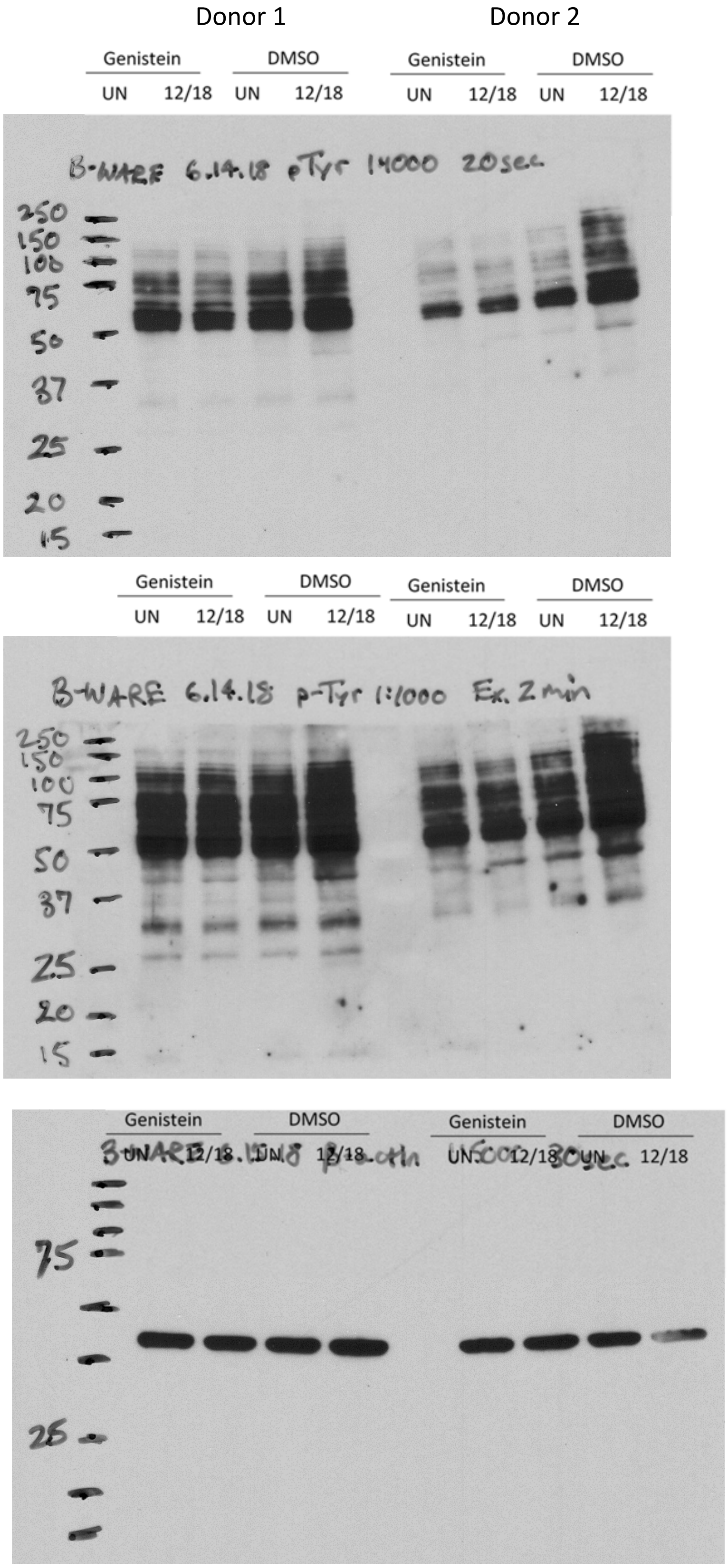

Donor 1 | Donor 2

Genistein | DMSO | Genistein | DMSO

UN | 12/18 | UN | 12/18 | UN | 12/18 | UN | 12/18

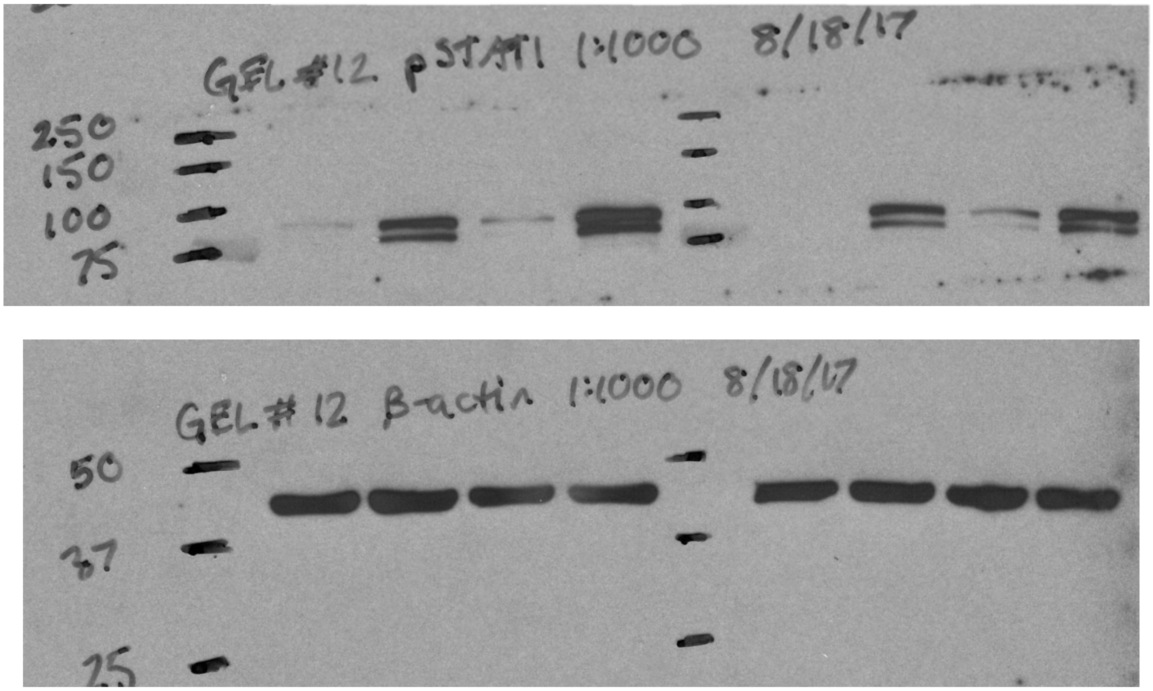

Genistein | DMSO | Genistein | DMSO

UN | 12/18 | UN | 12/18 | UN | 12/18 | UN | 12/18

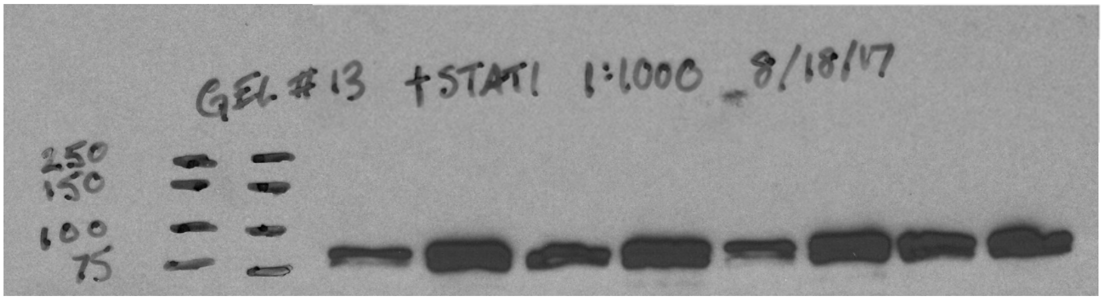

Donor 3

Genistein		DMSO	
UN	12/18	UN	12/18

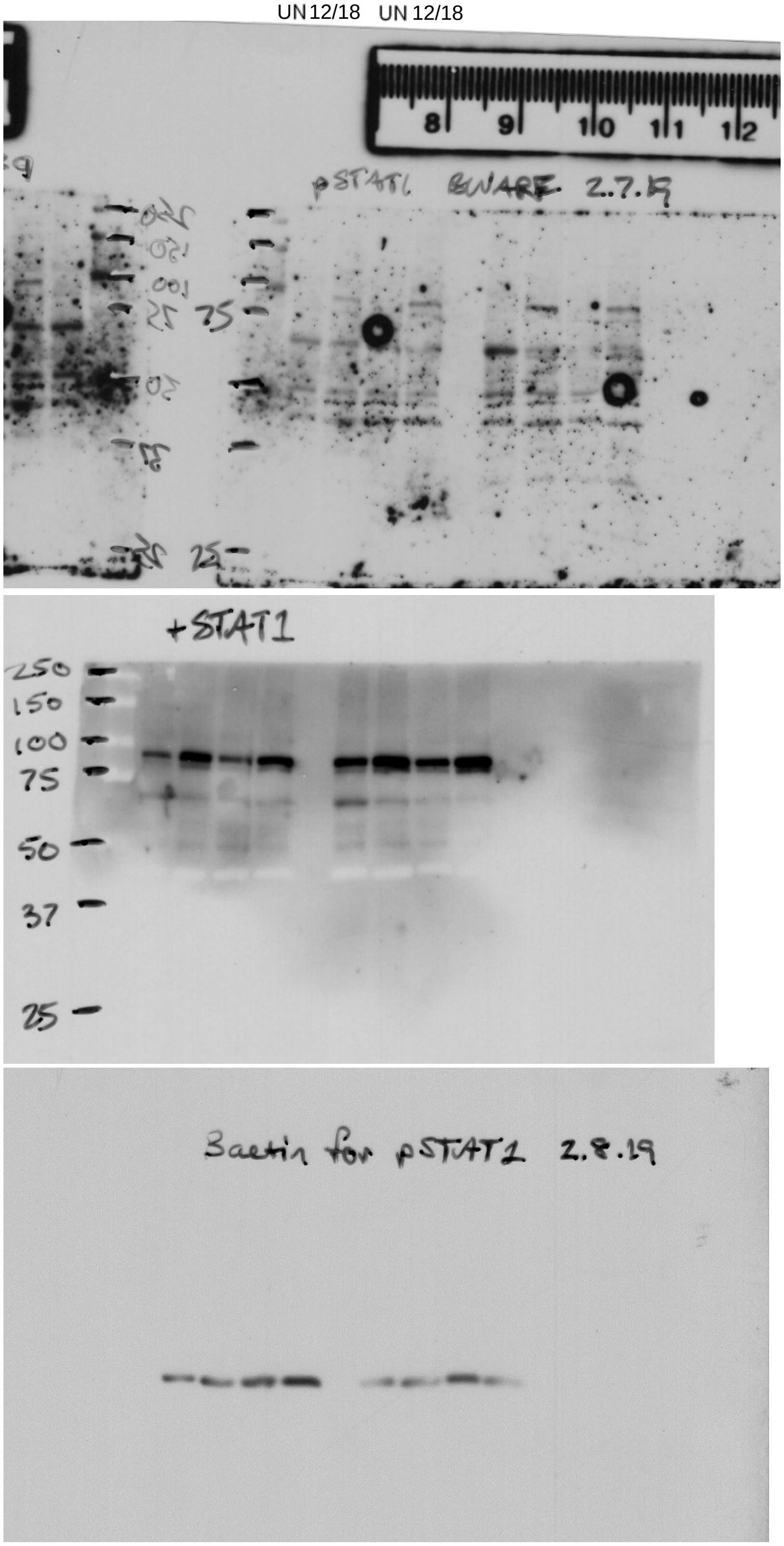

Donor 3

Genistein DMSO

UN 12/18 UN 12/18

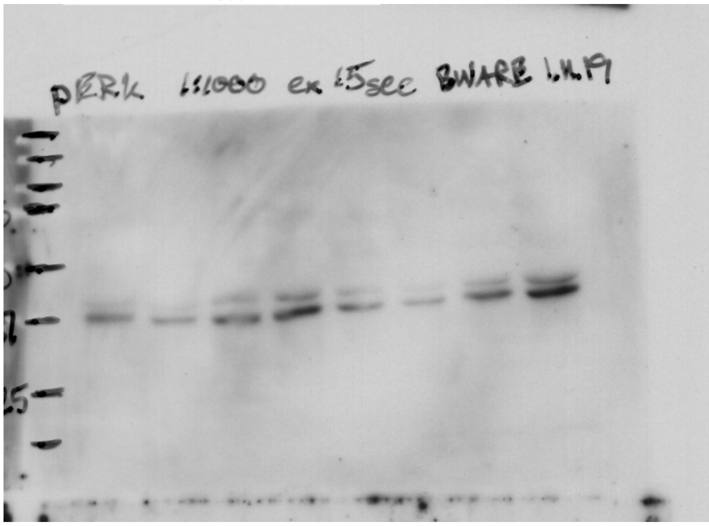

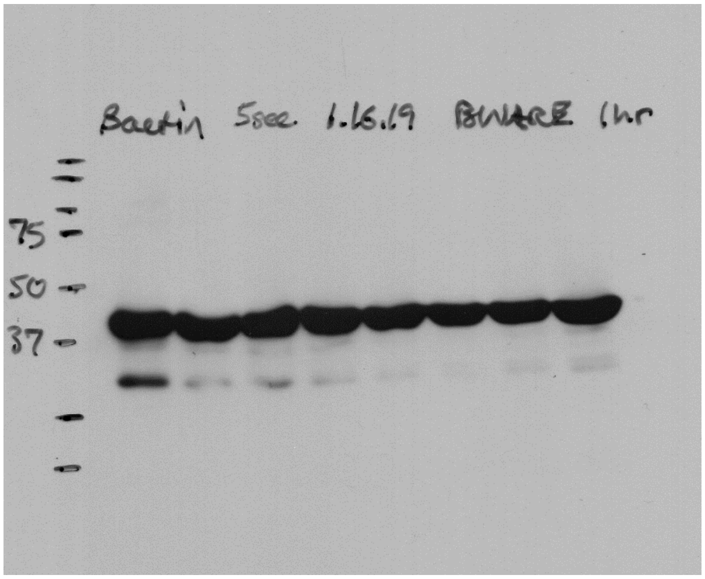

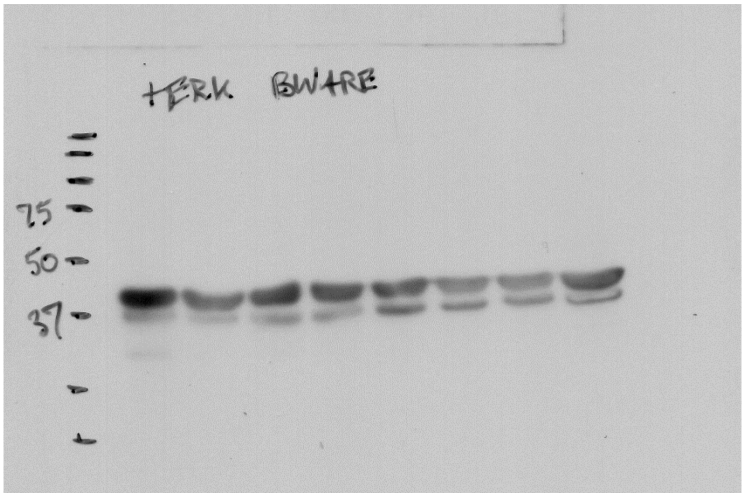

Donor 3

Genistein DMSO

UN 12/18 UN 12/18

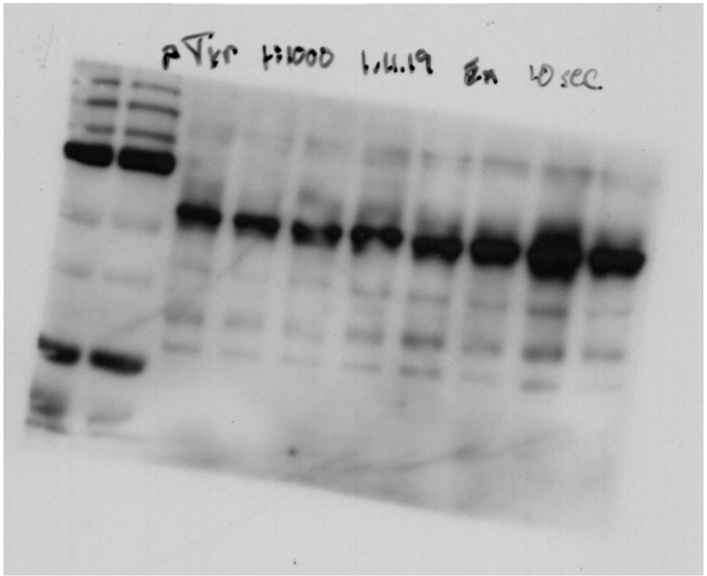

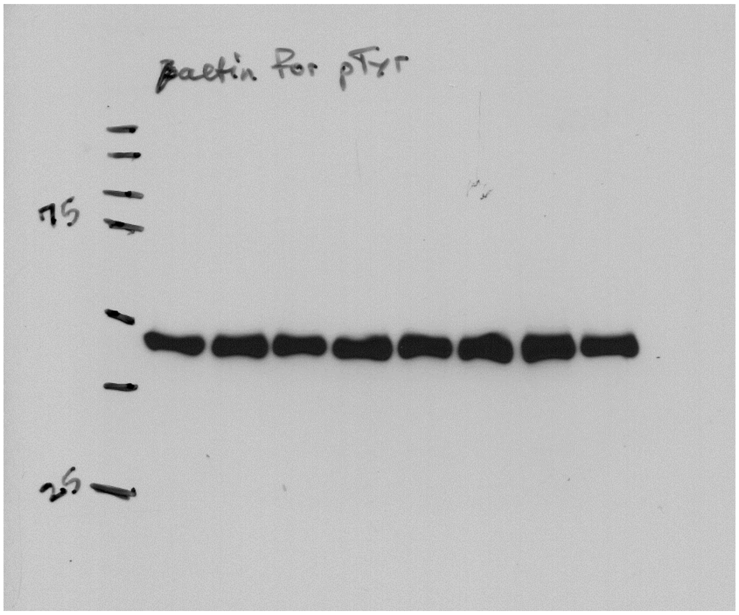

Supplement: Supplementary file 1 — Supplemental Data All [file 41598_2019_41687_MOESM1_ESM.pdf]
